# New insights on the regulation of *INK4/ARF* locus expression

**DOI:** 10.18632/oncotarget.22258

**Published:** 2017-11-01

**Authors:** Cristina Gamell, Doron Ginsberg, Sue Haupt, Ygal Haupt

**Affiliations:** Cristina Gamell: The Sir Peter MacCallum Department of Oncology, The University of Melbourne, Melbourne, Australia; Tumor Suppression Laboratory, Peter MacCallum Cancer Centre, Melbourne, Australia

**Keywords:** E6AP, INK4/ARF, ANRIL, NSCLC, E2F1

The *INK4/ARF* locus encodes the key tumor suppressors, p15^INK4b^, p16^INK4a^ and ARF, that together regulate the pRb and p53 major tumor suppressive networks. Given the importance of the products of the *INK4/ARF* locus in tumor suppression, transcription from the locus is tightly governed. Over the last decade, intense study to elucidate the regulation of this locus exposed that the genes encoded in the *INK4/ARF* locus are controlled both independently as well as in a coordinated manner.

**Figure 1:**
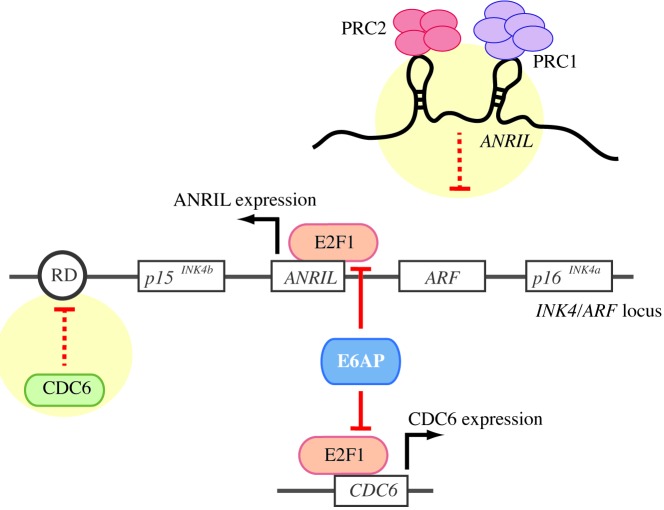
Schematic model illustrating the molecular mechanism proposed for E6AP stimulation of the expression of the genes encoded in the *INK4/ARF* locus

A known regulatory site responsible for the coordinated silencing of all three genes in the *INK/ARF* locus is positioned in close proximity to the p15^INK4b^ gene called Regulatory Domain (RD). Interaction of the CDC6 protein with the RD triggers recruitment of histone deacetylases, which increase methylation of histone H3 on lysine 9 (H3K9) and in turn cause heterochromatinization of the p15^INK4b^, p16^INK4a^ and ARF promoters leading to their silencing. In this context, we recently reported that the E3 ubiquitin ligase and transcription cofactor E6AP (also known as UBE3A) acts as a positive regulator of the *INK4/ARF* locus [1]. Mechanistically, we demonstrated that E6AP inhibits CDC6 expression by interacting with E2F1 and reducing its ability to bind to the CDC6 promoter and induce its transactivation (Figure 1). Importantly, this novel regulatory pathway is clinically relevant in non-small cell lung cancer (NSCLC), since we showed that patients with the E6AP-low/CDC6-high/ p16^INK4a^-low expression endure the worst overall survival. This correlation predicts the prognostic value of this expression signature.

A key finding from our work is that the ability of E6AP to modulate E2F1 transcriptional activity is not limited to the *CDC6* gene, as we demonstrated that the levels of other E2F1 gene targets were also altered in E6AP-deficient cells. In accord with these findings, we recently published an independent report showing that in prostate cancer cells, E6AP reduces the ability of E2F1 to bind to p27 promoter and to induce its transcription [2].

A particularly intriguing regulator of the expression of the tumour suppressors encoded in the *INK4/ARF* locus is the long non-coding RNA (lncRNA) *ANRIL* (antisense non-coding RNA in the INK4 locus). *ANRIL* gene is transcribed in the antisense orientation relative to the *INK4/ARF* gene cluster. *ANRIL* promotes the epigenetic repression of its neighbor tumor suppressors in the *INK4/ARF* locus by physically interacting with the Polycomb proteins Suz12 and Cbx7, which in turn recruit the Polycomb repressive complex 1 and 2 to the locus, leading to histone modification and silencing of the locus [3].

The contribution of *ANRIL* to cancer development has attracted much attention in recent years, and *ANRIL* oncogenic role is now solidly established in a variety of carcinomas. This is the case for NSCLC, where multiple recent papers convincingly demonstrate that high *ANRIL* levels correlate with poor prognosis (i.e. [4]). In this context however, very little is known about the mechanisms of *ANRIL* regulation. The only hints come from two independent groups that reported that *ANRIL* is transcriptionally activated by E2F1 [5, 6]. This finding, together with our recently published data showing that E6AP regulates the expression of the *INK4/ARF* locus, and that E6AP modulates E2F1 transcription activity, prompt us to speculate that E6AP could maintain expression levels of the tumor suppressor genes in the *INK4/ARF* locus by inhibiting *ANRIL* in an E2F1-dependent manner. We propose a model wherein E6AP promotes activation of the *INK4/ARF* locus by repressing the expression of the two key negative regulators of the locus, CDC6 and *ANRIL*. Further studies will be required to establish the relevance of the E6AP/E2F1/*ANRIL*/ p16^INK4a^ axis in NSCLC, as well as in other cancers. Nevertheless, we believe this opens potential new therapeutic options in the light of the recent interest that therapeutics based on lncRNAs targeting are attracting in the context of cancer [7].

In summary, our paper by Gamell *et al.* raises another potential regulatory mechanism of the *INK4/ARF* locus that is relevant in NSCLC, and opens new potential avenues for identification of novel pathways in NSCLC pathogenesis. An interesting future frontier will be to integrate all these pathways involved in the regulation of the *INK4/ARF* products to design better treatment strategies to ultimately improve outcomes of cancer patients.
